# Clinician dashboard views and improvement in preventative health outcome measures: a retrospective analysis

**DOI:** 10.1186/s12913-019-4327-3

**Published:** 2019-07-11

**Authors:** Patrick A. Twohig, Jaclyn R. Rivington, Douglas Gunzler, Joseph Daprano, David Margolius

**Affiliations:** 1 Department of Medicine, Case Western Reserve University, MetroHealth System, 2500 MetroHealth Drive, Cleveland, OH 44109 USA; 2 Center for Health Care Research and Policy, Case Western Reserve University, 2500 MetroHealth Drive, Cleveland, OH 44109 USA; 3 Department of Medicine-Pediatrics, Case Western Reserve University, MetroHealth System, 2500 MetroHealth Drive, Cleveland, OH 44109 USA

**Keywords:** Clinical dashboards, Quality improvement, Colorectal cancer, Screening, Hemoglobin A1c

## Abstract

**Background:**

Measuring and reporting outcome data is fundamental for health care systems to drive improvement. Our electronic health record built a dashboard that allows each primary care provider (PCP) to view real-time population health quality data of their patient panel and use that information to identify care gaps. We hypothesized that the number of dashboard views would be positively associated with clinical quality improvement.

**Methods:**

We performed a retrospective analysis of change in quality scores compared to number of dashboard views for each PCP over a five-month period (2017–18). Using the manager dashboard, we recorded the number of views for each provider. The quality scores analyzed were: colorectal cancer (CRC) screening rates and diabetic patients with an A1c greater than 9% or no A1c in the past year.

**Results:**

Data from 120 PCPs were included. The number of dashboard views by each PCP ranged from 0 to 222. Thirty-one PCPs (25.8%) did not view their dashboard. We found no significant correlation between views and change in quality scores (correlation coefficient = 0.06, 95% CI [− 0.13, 0.25] and − 0.05, 95% CI [− 0.25, 0.14] for CRC and diabetes, respectively).

**Conclusion:**

Clinical dashboards provide feedback to PCPs and are likely to become more available as healthcare systems continue to focus on improving population health. However, dashboards on their own may not be sufficient to impact clinical quality improvement. Dashboard viewership did not appear to impact clinician performance on quality metrics.

## Background

Measuring and reporting outcome data are keys for health care systems to identify opportunities to improve care [1]. One proposed method of achieving this is using dashboards [2]. Dashboards are associated with reduced length of stay, timeliness of discharges, improved patient outcomes, increase grant funding, and staff participation when end-user input is incorporated [3–5]. Dashboards have the potential to guide program development, mobilize healthcare providers to improve care, and demonstrate program value to stakeholders [6]. Clinical dashboards are designed to display data to clinicians that impact quality of care [1]. Our health care system partnered with our electronic health record (EHR), Epic Systems, to build a clinical dashboard that allows each primary care provider (PCP) to view quality data of their primary care panel and reveal the registry of their patients with a care gap for each metric for a limited number of chronic diseases and preventive cancer quality measures. A manager dashboard displayed the number of times each PCP viewed the clinical dashboard. We report the correlation of dashboard views and quality improvement. We hypothesized that clinical dashboard views lead to the providers having real-time feedback and thus more views would be associated with greater improvement in quality.

## Methods

**Fig. 1:**
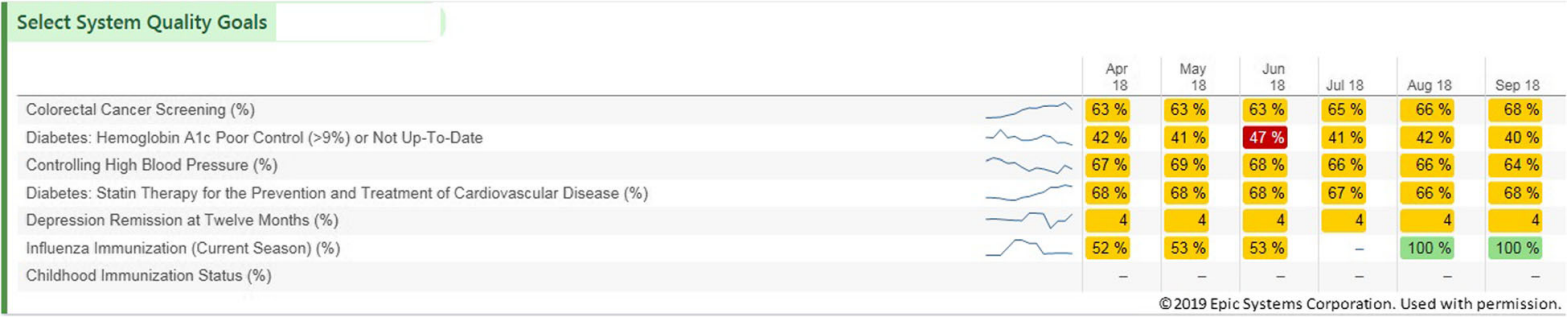
Screenshot of the clinical dashboard used in the EPIC Systems Electronic Medical Record showing select system quality metrics (left). Percentages indicate what proportion of a provider’s patient panel is up to date on that particular metric in each month of the year (right). Figure presented with permission from EPIC Systems©

We performed a retrospective analysis of change in quality scores compared to dashboard views for each PCP. The clinical dashboard was accessible with one click in the EHR, providing both individual and population level data (Fig. 1). To represent quality, we chose colorectal cancer (CRC) screening rates and percentage of patients with diabetes who had an A1c greater than 9% or no A1c in the past year. To be up to date on colorectal cancer screening, patients needed to have had a colonoscopy in the last 10 years or a fecal immunochemical test done in the last one year. We chose these two metrics out of many possibilities because they were clinically meaningful, able to be impacted by clinicians, aligned with value-based contracts such as our Medicare Accountable Care Organization, and we perform below national benchmarks as a system. We recorded the numerator and denominator for each PCP’s metric in June 2017 and January 2018 (Table 1). The numerator indicates the number of patients who were up to date (for CRC screening) or who had a care gap (for the diabetes metric). The denominator equals the number of patients in their panel who were eligible for the metric. A panel consisted of the total number of patients assigned to the provider in the EHR.

**Table 1:** Change in quality scores for CRC and A1c > 9%

		June 2017	January 2018
Colorectal Cancer	Numerator	27,491	31,576
	Denominator	50,515	53,671
	Percentage	54.42%	58.83%
Diabetes A1c > 9% or Not Up to Date	Numerator	6,100	6,201
	Denominator	17,390	18,446
	Percentage	35.08%	33.62%

We recorded the number of views between September 2017 (when the data was first available) and January 2018 for each provider. We chose to conclude our study window in January because in February 2018, clinician revenue productivity data was added to the dashboard which we felt would confound our dashboard views measure. Clinicians received monthly e-mail instructions for how to use the clinical dashboard and all clinicians attended an in-person training session during a retreat. Concrete suggestions for how to improve quality were included in newsletters but not on the clinical dashboard. PCPs were included if they had a panel at the beginning and end of the time interval. Trainees and the authors were excluded. The study was approved by the MetroHealth Institutional Review Board.

We calculated Spearman’s rank correlation coefficients between dashboard views and change in quality score. We used a generalized estimating equations (GEE) approach with a logit link function to account for differences in the raw counts (numerator and denominator) and changes in percentage. Due to the extreme skewness of the distribution of dashboard views, we performed a log (dashboard views+ 1) transformation for this analysis.

## Results

Data from 120 PCPs were included. The number of dashboard views by each PCP ranged from 0 to 222 views in the 5-month interval (mean 14, median 2). Thirty-one PCPs (25.8%) did not view their dashboard. Baseline quality scores were highly variable between providers.

We found no significant correlation between views and change in quality scores (for CRC screening: correlation coefficient = 0.06, 95% CI [− 0.13, 0.25]; for diabetes: correlation coefficient = − 0.05, 95% CI [− 0.25, 0.14]). Likewise, the GEE procedure revealed no statistically significant correlation. Table 1 shows the total number and percentage of patients who were up to date (for CRC screening) or who had a care gap (for the diabetes metric) between June 2017 and January 2018. The was an overall improvement in the percentage of patients who were up to date for CRC screening (54.42 to 58.83%). Similarly, there was a decrease in the number of patients with a care gap or an A1c > 9% from June to January (35.08% vs. 33.62%).

## Discussion

Our findings show that adding a dashboard alone may not improve quality. Audit and feedback techniques have been shown to improve quality outcomes but depend on how the feedback is provided [2, 7]. Clearer targets, concrete suggestions, and combined oral and written feedback may have increased dashboard efficacy. Monthly during the intervention, we emailed reminders to clinicians on how to access their dashboard and suggestions for how to improve colorectal cancer screening and diabetes control. Our intervention fell short of providing timely, non-punitive feedback to providers with specific action items [7].

One limitation of our study was that the dashboard views were specific to provider views: we did not analyze the effect of views by practice managers, nurses, and other team members on quality improvement. Broader viewership may have enhanced the ability for care teams to improve quality data by contacting patients and addressing care gaps. A second important limitation was that six months may be too short a time to expect improved clinical quality.

## Conclusion

As a stand-alone intervention, dashboards may not be sufficient for impacting clinician performance on quality metrics; however, using a multi-faceted approach which includes clinician dashboards may be an important ingredient for successful quality improvement efforts [1, 8]. Future studies should evaluate program implementation over longer periods of time and with a wider outcome assessment that incorporates more organizational personnel and stakeholders at multiple levels of health care provision [9]. Hopefully others can learn from our missing components for using dashboards to drive improvement.

## Data Availability

The datasets used and/or analysed during the current study are available from the corresponding author on reasonable request.

## Abbreviations

CRC: Colorectal cancer
EHR: Electronic health record
GEE: Generalized estimating equations
PCP: Primary care provider
